# LEADING ADVANCE CARE CONVERSATIONS: DEVELOPMENT OF AN INTERVENTION PLATFORM

**DOI:** 10.1093/geroni/igad104.0154

**Published:** 2023-12-21

**Authors:** Kara Dassel, Nancy Aruscavage, Emily Nachtweih, Katherine Supiano, Rebecca Utz, Eli Iacob, Jordana Clayton

**Affiliations:** University of Utah College of Nursing, Salt Lake Cty, Utah, United States; University of Utah College of Nursing, Salt Lake City, Utah, United States; University of Utah, Salt Lake City, Utah, United States; University of Utah, Salt Lake City, Utah, United States; University of Utah, Salt Lake City, Utah, United States; University of Utah, Salt Lake City, Utah, United States; University of Utah College of Nursing, Salt Lake City, Utah, United States

## Abstract

The LEAD Guide (Life-Planning in Early Alzheimer’s and Other Dementias) is an advance care planning guide designed specifically for people with dementia and their care partner. It addresses changes in cognition and goals of care, including values and preferences, for end-of-life care and support across the disease continuum. The goal of using the guide is to create a shared understanding of the care recipient’s end-of-life care preferences. We developed an interactive web-based format of the LEAD Guide, an enhanced extension of the original paper guide, for a pilot behavioral intervention. The LEAD Intervention guided community-based Alzheimer’s disease and related dementias (ADRD) dyads (N=60 dyads; persons concerned, at risk, or with a dementia diagnosis and their care partners) through advance care planning (ACP) conversations and documentation. The intervention model included the LEAD Guide, interactive instructions, and educational material about ACP in the context of dementia. We developed the LEAD Intervention using an interactive design and beta testing process (including surveys and focus groups) with our community advisory board members. The board members included professional dementia experts, community partners, and ADRD dyads. Board members were enthusiastic about the LEAD Intervention and provided suggested revisions in three areas; technology (navigation of web-based documents, webpage design), personalization (the need to personalize emails and surveys to individual participants), and evaluation (creating simple scaled and open-text questions to obtain feedback on the intervention activities). Creating a feasible, acceptable, and efficacious intervention requires a multi-faceted approach that incorporates input from end users using community-based participatory research methods.

